# Interferon-α Promotes the Expression of Cancer Stem Cell Markers in Oral Squamous Cell Carcinoma: Erratum

**DOI:** 10.7150/jca.105108

**Published:** 2024-10-25

**Authors:** Hailong Ma, Shufang Jin, Wenyi Yang, Zhuowei Tian, Shuli Liu, Yang Wang, Ge Zhou, Mei Zhao, Shalva Gvetadze, Zhiyuan Zhang, Jingzhou Hu

**Affiliations:** 1 Department of Oral Maxillofacial-Head and Neck Oncology, Shanghai Ninth People's Hospital, Shanghai Jiao Tong University School of Medicine, Shanghai Key Laboratory of Stomatology, Shanghai 200011, China; 2 Department of Oral and Maxillofacial Surgery, The Second Affiliated Hospital Zhejiang University School of Medicine, Hangzhou 310009, China; 3 Department of Head and Neck Surgery, The University of Texas MD Anderson Cancer Center, Houston, Texas 77030, USA; 4 Central Research Institute of Dentistry and Maxillofacial Surgery, Congenital Maxillofacial Defects and Deformations, Timura Frunze 16, Moscow 119034, Russia

In the original version of our article, there was an error in Fig. 4A. Specifically, the representative image of 20 ng/m/ IFNα in HN30 cells in Figure 4A is incorrect. The correct image is provided below. This correction will not affect the results and conclusions. The authors apologize for any inconvenience this may have caused.

## Figures and Tables

**Figure 4:**
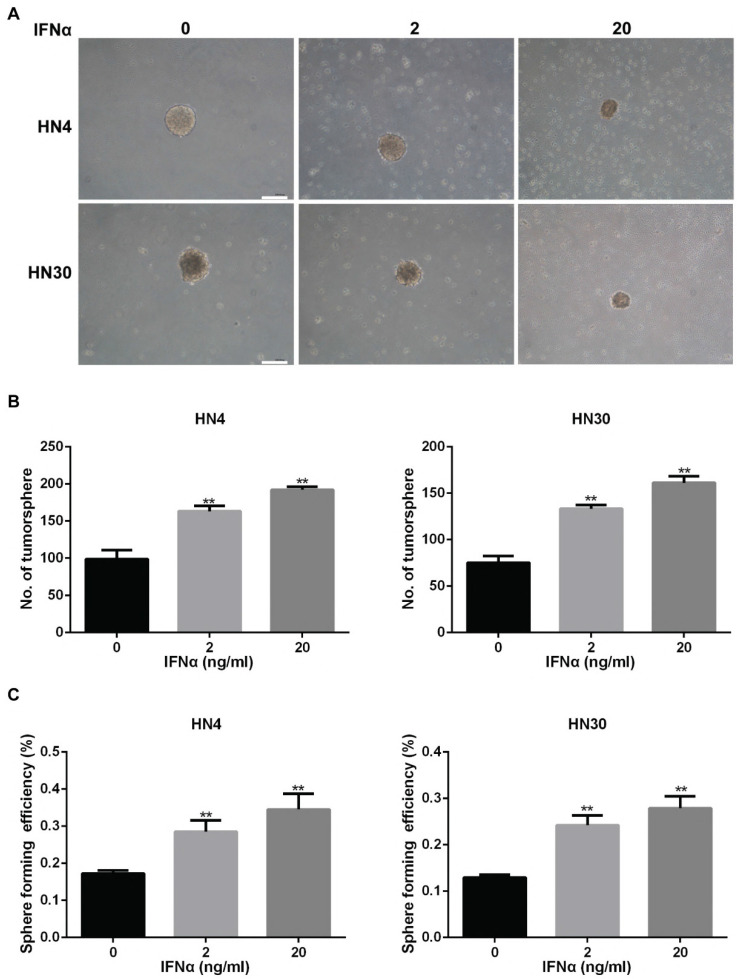
IFNα enhances tumorsphere formation ability in HN4 and HN30 cells. (A) Representative images of tumorsphere formation in conditioned medium with indicated concentration of IFNα (2ng/ml; 20ng/ml) for 2 weeks. Scar bar: 1mm. (B) Quantitation of the number of tumorsphere formation in HN4 and HN30 cells in the presence and absence of IFNα incubation. (C) Sphere forming efficiency of HN4 and HN30 cells in the presence and absence of IFNα incubation.

